# Trends in Gender and Blindness in India

**Published:** 2017-02-10

**Authors:** GVS Murthy, Hira Ballabh Pant, Souvik Bandyopadhyay, Neena John

**Affiliations:** 1 Vice-President, South, Public Health Foundation of India & Director, Indian Institute of Public

**Figure:**
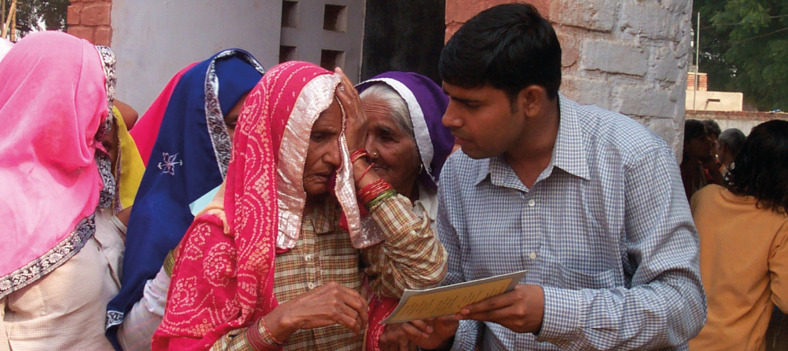
Women tend to have a higher rate of blindness with lesser access to health care.

Among the many definitions one that succinctly described equity is in a paper published in 2003.^1^ The authors defined equity in health as the absence of systematic disparities in health or the major social determinants of health between social groups who have different levels of underlying social advantages or disadvantages and which put people who are already socially disadvantaged at a further disadvantage with respect to their health.^1^ The underlying premise was that health is essential to wellbeing and to overcome other effects of social disadvantage.^1^

One of the social determinants of health that has been universally identified is gender. Health inequalities between men and women have been postulated to result from societal structures, role expectations and the cultural context.^2^,^3^ It has been emphasized that women bear a disproportionate burden of health inequity across the globe and face unique barriers in accessing health care.^4^ With respect to eye care, women are more likely to have higher rates of blindness and are less likely to access appropriate eye services.^5–8^ Available evidence points to a higher prevalence of blindness among women compared to men in all regions of the world after control ling for age as in South Asia, the age-standardized adult prevalence of blindness in women is 1.26 times the prevalence among male adults^9^.

India has been one of the countries where efforts to strengthen the evidence-base for blindness control has received significant attention from policy planners and program managers. Over the past four decades a series of population-based blindness and visual impairment surveys have been undertaken in India, using different survey methods. This included detailed eye examination surveys as well as rapid assessments.

To discern the temporal trends in relation to blindness and gender differentials we have used data from two large population-based surveys in India. One was conducted over the period 1999-2001 (detailed eye examination survey)^7^ and the other over the period 2006–2007 (rapid assessment of blindness survey).^8^ Both surveys looked at populations aged ≥ 50 years and defined blindness based on presenting vision (visual acuity < 3/60 in both eyes).

A total of 108,609 individuals were examined in the two surveys in India (63,432 in 1999-2001 and 45,177 in 2006–2007).

The prevalence of blindness in 1999-2001 was 5.36% [95% CI: 5.2-5.5] while in 2006-2007, it was 3.82% [95% CI: 3.64 – 4.0]. These results show that there was a significant reduction in the prevalence of blindness over this period. The prevalence of blindness amongst males was 4.19% [95% CI: 3.97-4.42] in 1999-2001 compared to 3.05% [95% CI:2.82-3.3] in 2006-2007 while in females it was 6.4% [95% CI: 6.14-6.67] in 1999-2001 and 4.44% [95% CI: 4.19 – 4.70] in 2006-2007. The results show that there is a significant reduction in overall blindness between 1999-2001 and 2006-2007 (X2-138.41; p < 0.001). The difference between males in the two rounds of the surveys was also statistically significant (X2-43.41; p < 0.001). The same was also true for females (X2-103.79; p < 0.001). At the same time the difference in the prevalence of blindness between males and females was statistically significant both in 1999-2001 (X2-152.11; p < 0.001) and in 2006 – 2007 (X2-57.96; p <0.001). The risk of blindness in females was 1.41 times higher compared to males in the urban areas, while in rural areas the risk was 1.51 times higher. After adjusting for age, place of residence (urban/rural) and the year of the survey, it was observed that females had a 1.76 times higher risk of blindness compared to males. These findings show that there is a clear cut gender disparity in the prevalence of blindness in India. If one looks at the percentage reduction in prevalence of blindness, it was seen that there was a 71% reduction in the overall prevalence of blindness among those aged ≥50 years over a span of 8 years. Amongst males the reduction was 72.8% compared to 69.4% among females over the same period. Cataract was the principal cause of blindness both in 1999-2001 and 2006-2007. It was observed that males had a 40% lower risk of being cataract blind compared to females in both rounds of the surveys. This is an important observation as cataract is a treatable cause of blindness and an important determinant of avoidable blindness. The higher load of cataract blindness in females over the 8 year period demonstrates inequity and suggests that interventions to improve access to cataract services in women have not been sufficient. In India where the overall status of women in society is poor, a gender focus is essential if gender equity is to be ensured, especially when access to services is poor. Exclusive special incentives like higher reimbursement for females operated compared to males or for non-monetary incentives like a certificate of ‘women-friendly institution’ etc., to operate on the females will help in enhancing access to women and thereby reduce the gender differentials. The situation is likely to be similar in countries of the South Asia region with similar economies to India.

**Table 1:** Prevalence of blindness and association with gender in India

Characteristics	1999-2001		2006-2007	
	**N (%)**	**Prevalence[95%CI]**	**N (%)**	**Prevalence [95%CI]**
**No. examined**	63,432	-	45,177	-
**No. males examined**	30,013	-	20,331	-
**No. female examined**	33,419	-	24,846	-
**Prevalence of Blindness**	5.36%	5.18 – 5.53	3.82%	3.64 – 4.0
**Prevalence of Blindness (Male)**	4.19%	3.97 – 4.42	3.05	2.82 – 3.30
**Prevalence of Blindness (Female)**	6.40%	6.14 – 6.67	4.43%	4.19 – 4.70
